# Correction: The Open Anchoring Quest Dataset: Anchored Estimates from 96 Studies on Anchoring Effects

**DOI:** 10.5334/jopd.92

**Published:** 2024-08-23

**Authors:** Lukas Röseler, Lucia Weber, Katharina Helgerth, Elena Stich, Miriam Günther, Paulina Tegethoff, Felix Stefan Wagner, Elitza Ambrus, Martina Antunovic, F. Barrera-Lemarchand, Eliran Halali, Konstantinos Ioannidis, Oliver Genschow, Ryan Mckay, Nir Milstein, D. C. Molden, Frank Papenmeier, Zoran Pavlović, Robin Rinn, Marie Luise Schreiter, M. F. Zimdahl, Eilish Allen, Bahník Štěpán, C. Bermeitinger, F. B. N. Blower, Hannah Luisa Bögler, P. Burgmer, Nathan N. Cheek, Linda Dorsch, Sabine Andrea Fels, Marie-Lena Frech, L. Freira, A. J. L. Harris, Björn Hartig, Jan A. Häusser, M. V. Hedgebeth, Maureen Henkel, D. Horvath, Roland Imhoff, Paula Intelmann, A. Klamar, Ella Knappe, L.-M. Köppel, Sabine Marie Krueger, S. Lagator, Florencia López Bóo, J. Navajas, J. K. Norem, Janina Novak, Y. Onuki, Elise Page, Jodie Pearton, Susanne Ponader, Tobias R. Rebholz, Adriana Rostekova, M. Sartorio, Sebastian Schindler, Christian Seida, D. R. Shanks, M.-C. Siems, Maarten Speekenbrink, P. Stäglich, Mara Starkulla, M. Stitz, Thomas Straube, K. Thies, Elias Thum, K. Ueda, M. Undorf, D. Urlichich, M. A. Vadillo, H. Wolf, A. Zhou, A. Schütz

**Affiliations:** 1 University of Bamberg, University of Münster, Münster Center for Open Science, Germany; 2 University of Bamberg, Germany; 3 Royal Holloway, University of London, United Kingdom; 4 University of Tübingen, Germany; 5 Universidad Torcuato di Tella, Argentina; 6 Universidad de Buenos Aires, Argentina; 7 Bar-Ilan University, Israel; 8 University of Amsterdam, Netherlands; 9 University of Cologne, Germany; 10 Leuphana University Lüneburg, Germany; 11 Northwestern University, United States; 12 University of Belgrade, Serbia; 13 University of Würzburg, Constructor University Bremen, Germany; 14 University of Mannheim, Germany; 15 Prague College of Psychosocial Studies, Czechia; 16 University of Hildesheim, Germany; 17 University College London, United Kingdom; 18 University of Southampton, United Kingdom; 19 Purdue University, United States; 20 Justus-Liebig-University Giessen, Germany; 21 Virginia Commonwealth University, United States; 22 University of Würzburg, Germany; 23 Technical University of Darmstadt, Germany; 24 Vienna University of Economics and Business, Wien, Austria; 25 Johannes Gutenberg University Mainz, Germany; 26 Federal University of Applied Administrative Sciences, Germany; 27 Harz University of Applied Science, Germany; 28 InterAmerican Development Bank, Washington DC, United States; 29 Wellesley College, United States; 30 The university of Tokyo, Japan; 31 University of Münster, Germany; 32 Universidad Autónoma de Madrid, Spain

**Keywords:** Anchor, anchoring-and-adjustment, assimilation, judgment and decision making, estimates

## Abstract

This article details a correction to: Röseler, L., Weber, L., Helgerth, K., Stich, E., Günther, M., Tegethoff, P., … Schütz, A. (2022). The Open Anchoring Quest Dataset: Anchored Estimates from 96 Studies on Anchoring Effects. *Journal of Open Psychology Data*, 10(1), 16. DOI: https://doi.org/10.5334/jopd.67

## Correction

The article, ‘The Open Anchoring Quest Dataset: Anchored Estimates from 96 Studies on Anchoring Effects’ (Röseler, et al., 2022) was mistakenly published with the following co-authors omitted: Elitza Ambrus, Ryan McKay, Bjoern Hartig, Eilish Allen, Adriana Rostekova, Jodie Pearton, Susanne Ponader, Roland Imhoff, Christian Seida, and Maarten Speekenbrink.

This was due to an administrative error.

All co-authors confirm that they meet the definitions of authorship and made substantial contributions to the paper.

## References

[1] Röseler, L., Weber, L., Helgerth, K., Stich, E., Günther, M., Tegethoff, P., … Schütz, A. (2022). The Open Anchoring Quest Dataset: Anchored Estimates from 96 Studies on Anchoring Effects. Journal of Open Psychology Data, 10(1), 16. DOI: 10.5334/jopd.67 PMC12270249 40687673

